# Use of postmenopausal hormone therapies and risk of histology- and hormone receptor-defined breast cancer: results from a 15-year prospective analysis of NIH-AARP cohort

**DOI:** 10.1186/s13058-020-01365-9

**Published:** 2020-11-25

**Authors:** Shao-Ming Wang, Ruth M. Pfeiffer, Gretchen L. Gierach, Roni T. Falk

**Affiliations:** 1 National Central Cancer Registry, National Cancer Center/National Clinical Research Center for Cancer/Cancer Hospital, Chinese Academy of Medical Sciences and Peking Union Medical College, Beijing, 100021 China; 2 Metabolic Epidemiology Branch, Division of Cancer Epidemiology & Genetics, National Cancer Institute, National Institutes of Health, 9609 Medical Center Dr. rm 6E344, Bethesda, MD 20892-9768 USA; 3 Biostatistics Branch, Division of Cancer Epidemiology & Genetics, National Cancer Institute, National Institutes of Health, Bethesda, MD USA; 4 Integrative Tumor Epidemiology Branch, Division of Cancer Epidemiology & Genetics, National Cancer Institute, National Institutes of Health, Bethesda, MD USA

**Keywords:** Breast cancer risk, NIH-AARP cohort, Menopausal hormone therapy

## Abstract

**Background:**

Menopausal hormone therapy (MHT) increases breast cancer (BC) risk, but cohort studies largely consider use only at enrollment. Evidence is limited on how changes in MHT use alter the magnitude of risk, and whether risk varies between invasive and in situ cancer, by histology or by hormone receptor status.

**Methods:**

We investigated the roles of estrogen-alone therapy (ET) and estrogen plus progestin therapy (EPT) on BC risk overall, by histology and estrogen receptor (ER) and progesterone receptor (PR) status, and on incidence of in situ disease, in the NIH-AARP cohort. Participants included 118,760 postmenopausal women (50–71 years), of whom 63.5% (*n* = 75,398) provided MHT use information at baseline in 1996 and in a follow-up survey in 2004, subsequent to the dissemination in 2002 of the Women’s Health Initiative trial safety concerns regarding EPT. ET analyses included 50,476 women with hysterectomy (31,439 with follow-up data); EPT analyses included 68,284 women with intact uteri (43,959 with follow-up data). Adjusted hazard ratios (HRs) were estimated using Cox proportional hazards models using age as the time metric with follow-up through 2011.

**Results:**

Eight thousand three hundred thirty-three incident BC cases were accrued, 2479 in women with follow-up data. BC risk was not elevated in current ET users at baseline (HR = 1.05, 95% confidence interval [CI] CI = 0.95–1.16) but was higher in women continuing use through 2004 (HR = 1.35, 95% CI = 1.04–1.75). Ever EPT use at baseline was associated with elevated BC risk overall (HR = 1.54 (1.44–1.64), with a doubling in risk for women with 10 or more years of use, for in situ disease, and across subtypes defined by histology and ER/PR status (all *p* < 0.004). Risk persisted in women who continued EPT through 2004 (HR = 1.80, 95% CI = 1.39–2.32). In contrast, no association was seen in women who discontinued EPT before 2004 (HR = 1.14, 95% CI = 0.99–1.30).

**Conclusions:**

ET use was not associated with BC risk in this cohort, although excess risk was suggested in women who continued use through 2004. EPT use was linked to elevated in situ and invasive BC risk, and elevated risk across invasive BC histologic and hormone receptor-defined subtypes, with the highest risk for women who continued use through the 2004 follow-up survey.

**Supplementary information:**

The online version contains supplementary material available at 10.1186/s13058-020-01365-9.

## Background

The relationship between MHT and postmenopausal breast cancer (BC) risk has been extensively studied [1, 2]. A recent meta-analysis of observational studies, which included over 100,000 women with invasive BC, concluded that all MHT formulations except vaginal creams were associated with excess risk, with stronger effects for estrogen plus progestin therapy (EPT) formulations than estrogen-alone therapy (ET) [2]. In contrast, the Women’s Health Initiative (WHI), the largest long-term randomized clinical trial of combined equine estrogens alone (CEE alone) or EPT, found a significantly higher risk of developing BC among women using EPT [3], while a lower risk was observed in the CEE alone arm [4]. Reasons for differences between the WHI and observational studies may be due in part to different population characteristics including body mass index (BMI) distribution, frequency of mammography screening, ages at initiation of MHT, and different therapeutic hormone formulations or dosing regimens [1, 5]. Additionally, whereas WHI restricted the CEE alone arm to postmenopausal women with hysterectomy and EPT to women with intact uteri, similar restrictions were not applied in most observational studies including the recent meta-analysis [2]; rather, for the 24 participating prospective cohorts, a nested case-control study design was implemented, with up to 4 randomly selected controls matched to each BC case based on age, year of birth, and region. Finally, for both cohort and randomized trial studies, incomplete capture of MHT use over the follow-up period or during the trial may bias risk estimates [6]. Most cohort studies only collect information at the time of enrollment, which likely does not reflect use over follow-up [6]; this was circumvented in the recent meta-analysis, where women were considered current MHT users only if their last update on hormone use was less than 5 years from the date of diagnosis of their matched case (their index date). In the WHI trial, lack of adherence to treatment protocol was notable, as 42% in the EPT arm discontinued use, while 10.7% in the placebo arm started this treatment [4].

Included in the recent meta-analysis of observational studies were data from the NIH-AARP Diet and Health Study with follow-up through June 2002 (*n* = 3657 breast cancers) [7]. In our prior analysis of these data, we found a significantly elevated BC risk for EPT, with a weaker effect for ET alone users [7]. Herein, we report findings from an extended follow-up of the NIH-AARP cohort through 2011, which was not included in the recent meta-analysis, in which an additional 4700 incident BCs were ascertained. Additionally, included in the present analysis are updated MHT usage patterns from a follow-up survey conducted in 2004, following the 2002 release of safety concerns from the WHI regarding EPT which led to a dramatic decline in MHT prescriptions. From the more than 8000 BC cases identified through 2011 (1062 in situ, 7271 invasive), we analyzed the effects of ET and EPT use by hysterectomy status on risk of subsequent in situ and invasive BC and BC subtypes characterized by histology and hormone receptor status. For the subset of participants completing the 2004 survey, we also explored whether the BC risk was altered with updated information on MHT use.

## Methods

### Study population

The NIH-AARP cohort was established in 1995–1996 with the mailing of questionnaires to 3.5 million AARP members, aged 50–71 years, living in one of six US states (California, Florida, Louisiana, New Jersey, North Carolina, and Pennsylvania) or one of two metropolitan areas (Atlanta, GA, and Detroit, MI) [8]. The baseline questionnaire (BQ) queried about demographic characteristics, dietary intake, and health-related behaviors. Overall, a total of 617,119 men and women (17.6%) returned the baseline questionnaire, with 567,169 (16.2%) satisfactorily completing it. In 1996–1997, a second risk factor questionnaire (RFQ) collected additional information on diet, family history of cancer, anthropometry, physical activity, and MHT use, with a 59.4% response rate (*n* = 337,071). For our analyses, MHT information collected from the RFQ was considered baseline use. After excluding participants who died (*n* = 1619), moved out of the study area before the second survey (*n* = 547), and were male (*n* = 188,115), a total of 146,790 women were identified. We further excluded women with proxy responses for the BQ (*n* = 6959) or RFQ (*n* = 3424), who reported a history of cancer other than non-melanoma skin cancer on either questionnaire (*n* = 9071), who reported extreme values (defined as > 2 interquartile ranges above the 75th percentile or below the 25th percentile of log-transformed values) for caloric intake (*n* = 1058) or BMI (*n* = 834), who were missing all information on MHT use (*n* = 698), who had no follow-up (*n* = 14), who were premenopausal (*n* = 5327), or who were missing information on hysterectomy status (*n* = 645). The final study population included 118,760 women (Supplementary Fig. 1). The study was approved by the Institutional Review Board of the US National Cancer Institute.

### Exposure assessment

#### Baseline risk factor questionnaire

The RFQ collected detailed data on MHT use through enrollment in 1997, including dates of first and last use, total duration of use, regimen (cyclic or continuous), usual dose, and formulation used for the longest period for both estrogens and progestins. For the initial analysis, current usage was defined by patterns at the time of the RFQ (i.e., baseline). ET analyses were restricted to postmenopausal women with a hysterectomy and who chose to use Premarin for the longest period of time. EPT analyses were limited to women with intact uteri at baseline; of these, women were considered EPT users if they used combined EPT formulations or used both estrogen and progestin, provided the dates of first use were within 90 days of each other or the durations of use for both formulations were identical. We further classified EPT users based on progestin dose being sequential (for fewer than 15 days per cycle) or continuous (every day of the cycle).

#### Follow-up questionnaire

In 2004, a questionnaire was administered to update MHT use in the prior 10 years, including date of last use, duration, regimen, and formulation of estrogens and progestins. Among the 118,760 eligible women, 80,251 (67.6%) returned the follow-up questionnaire (FUQ). After excluding those with illogical responses or incomplete information (*n* = 4853), a total of 75,398 (63.5%) women were included in the restricted sub-cohort (Additional file 1: Fig. S1). For this analysis, current usage was defined by patterns at the time of administration of the 2004 questionnaire. ET analyses were restricted to hysterectomized women reporting no MHT use or who used estrogen supplements for the longest period in both RFQ and FUQ, and EPT analyses to women with intact uteri at baseline who did not use MHT or reported EPT use in both RFQ and FUQ or used EPT in the RFQ but only progestins for the past 10 years, based on the FUQ. We further excluded women who moved out of the catchment area or had a BC diagnosis before the 2004–2005 FUQ survey, including 2825 women (1208 BC cases) for ET analysis and 3963 women (2030 BC cases) for EPT analysis. Thus, a total of 28,614 women (including 940 BC cases) were included for ET analysis, and 39,996 women (including 1539 BC cases) were included for EPT analysis (Additional file 2: Table S1).

### Ascertainment of outcomes

Vital status was obtained by linkage to the National Death Index, and cancer diagnoses were updated via linkage to cancer registries in the 8 states in the cohort, as well as in 3 nearby states to which participants tended to move (Arizona, Nevada, and Texas). Incident in situ and invasive BCs were identified by the behavior and histologic codes of the International Classification of Diseases for Oncology, Third Edition (ICD-O-3) [9]. We classified invasive (behavior code 3) BC into ductal (IDC, 8500/3 or 8523/3), lobular (ILC, 8520/3 or 8524/3), mixed ductal/lobular tumors (8522/3), and other subtypes. Ductal carcinoma in situ (DCIS) and lobular carcinoma in situ (LCIS) were defined using the same histology codes and behavior code 2. BC estrogen receptor (ER) and progesterone receptor (PR) status were reported by cancer registries and coded as described in the American Joint Committee on Cancer’s Collaborative Staging Site-Specific Factors Manual. For analyses on ER/PR, we restricted the study to invasive BCs only.

### Statistical analysis

We used Cox proportional hazards regression with age as the time scale and included age at cohort entry in the models to estimate HRs and 95% CIs of developing BC. For the full cohort analysis, follow-up began at the age at which the 1996 RFQ was returned. For the restricted sub-cohort, follow-up began at the age at which the 2004 FUQ was returned. Follow-up ended at the age at the earliest of the following events: movement out of the defined catchment area, death, BC diagnosis, or the end of follow-up (December 31, 2011).

Potential risk factors for BC (Additional file 2: Table S2) were assessed, with the final models adjusted for race/ethnicity, ages at first birth and menopause, alcohol consumption, BMI, physical activity levels, number of breast biopsies, family history of BC in a first-degree relative, and number of mammograms in the 3 years preceding the baseline 1996 questionnaire.

We assessed risk of BC overall and by subtypes defined by histology or ER/PR status. Linear trend tests were assessed in multivariable-adjusted models by considering duration and dose of MHT as continuous variables. Time since last use was considered a time-dependent variable in our models. For the restricted sub-cohort with FUQ data, we assessed BC risk by ever use, recency, total duration through 2004 (assessed by start and stop times in the RFQ and FUQ), and time since last use, for ET and EPT use, respectively, with MHT analyzed as a time-varying exposure. Thus, a woman could have been a user at the start of the study but stop and start MHT use again prior to the FUQ.

Several sensitivity analyses were conducted including analyses stratified by BMI and by BC tumor subtypes defined by histology and hormone receptor status separately for ER and PR. Analyses were conducted using SAS, version 9.4 (SAS Institute, Inc., Cary, NC). Statistical tests were 2-sided, and *p* values < 0.05 were considered statistically significant. Figures were produced using the R forestplot package (version 3.4.2).

## Results

Between 1996 and 2011, a total of 8333 incident BC cases (6733 invasive) were identified among 118,760 women during a total of 1.53 million person-years of follow-up (median = 15.1 years, IQR 12.8–15.1 years). ER/PR status data were available for 69.5% (*n* = 4682) of invasive BC cases. Mean age at entry was 62.9 years, and the majority were non-Hispanic White (91.3%). The distribution of BC cases by race/ethnicity is shown (Additional file 2: Table S1). Distributions of potential risk factors from the baseline questionnaire are shown for MHT users and non-users, separately for women with hysterectomy and intact uteri, in Additional file 2: Table S2. Among women with hysterectomy, 20.1% never used MHT, 47.3% used conjugated equine estrogens (herein considered ET users), 10.5% reported other types of estrogens, and the remaining 22.1% reported having used various MHT formulations over time, including progestins (e.g., PT followed by EPT, ET followed by PT, PT followed by ET, EPT followed by PT, EPT followed by ET, EPT unknown, other combinations or unknown). Among women with intact uteri, 51.5% never used HT and 32.5% reported EPT use (either EPT alone or EPT use preceded by ET, with both types of users having similar risks). The remaining women (16%) used various MHT formulations as discussed for hysterectomized women. More than 90% of women in the ET group reported a surgical menopause while almost all women (98.5%) in the EPT analysis group had a natural menopause. For this analysis, the focus was as follows: for women with hysterectomy, we compared never MHT users (*n* = 10,120, including 615 BCs) to users of ET (*n* = 23,893, including 1489 BCs), and for women with intact uteri, women with no MHT use (*n* = 35,140, including 2170 BCs) were the referent for EPT users (*n* = 22,211, including 2110 BCs).

**Fig. 1:**
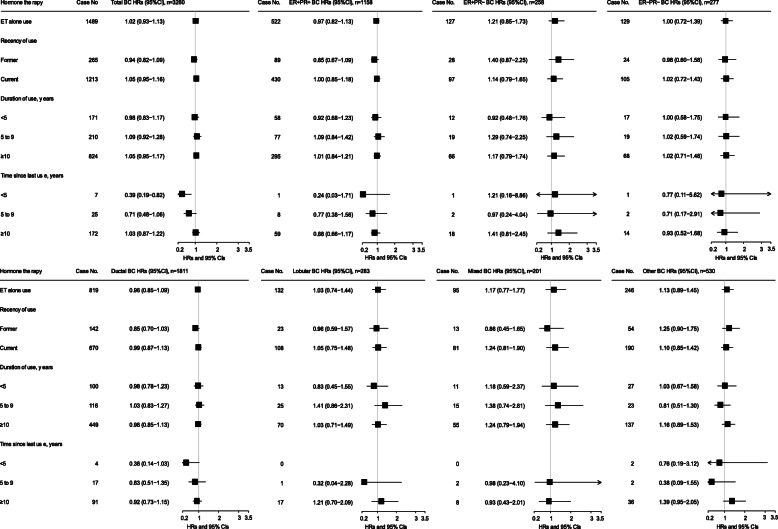
Associations between estrogen-alone therapy (ET) at baseline and risk of breast cancer, by estrogen and progesterone receptor status and histology type, among eligible postmenopausal women with hysterectomy (*n* = 50,476) in the entire National Institutes of Health-AARP Diet and Health Study cohort (1996/1997–2011). BC, breast cancer. ER, estrogen receptor. PR, progesterone receptor

**Fig. 2:**
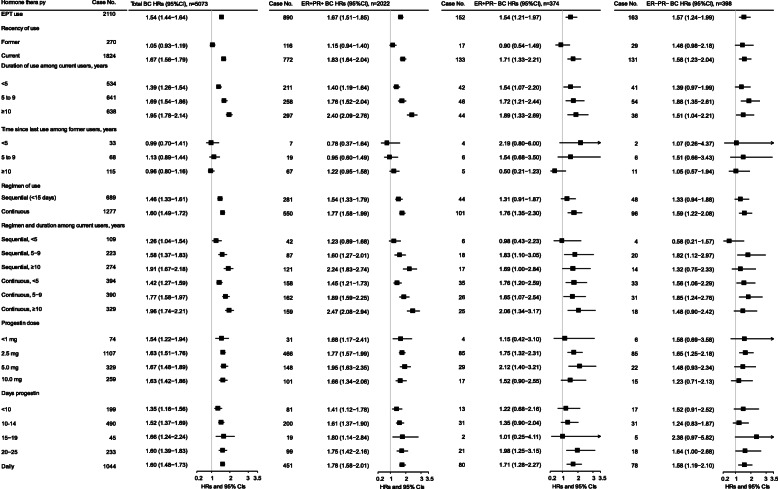
Associations between estrogen plus progestin therapy (EPT) at baseline and risk of breast cancer, by estrogen and progesterone receptor status, among eligible postmenopausal women with intact uteri (*n* = 68,284) in the entire National Institutes of Health-AARP Diet and Health Study cohort (1996/1997–2011). BC, breast cancer. ER, estrogen receptor. PR, progesterone receptor

**Fig. 3:**
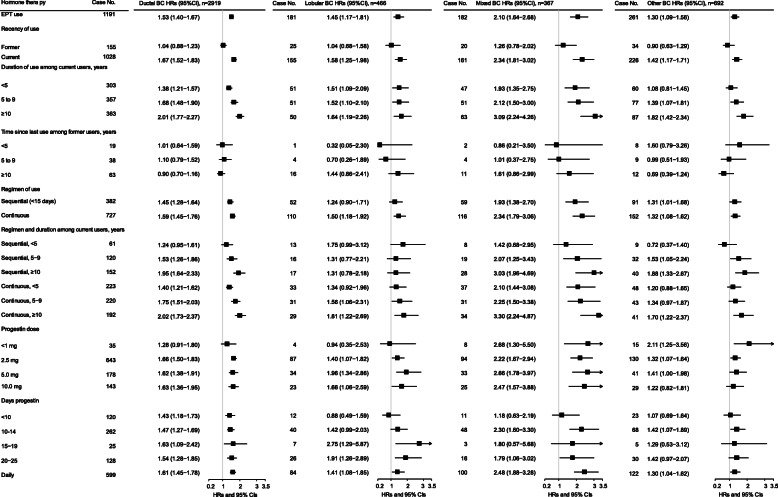
Associations between estrogen plus progestin therapy (EPT) at baseline and risk of breast cancer by histology type among eligible postmenopausal women with intact uteri (*n* = 68,284) in the entire National Institutes of Health-AARP Diet and Health Study cohort (1996/1997–2011). BC, breast cancer. ER, estrogen receptor. PR, progesterone receptor

Risks for ET use at baseline (not accounting for changes in use documented in the follow-up survey) are shown in Fig. 1 and Additional file 2: Tables S3, S4, and S5 and for EPT use in Figs. 2 and 3 and Additional file 2: Tables S3, S6, and S7. Results are for BC risk overall and for subtypes defined by ER/PR status and histology. For baseline ET use, there was no association with BC risk overall or by histologic or ER/PR subtypes (Fig. 1). For EPT at baseline, ever use was associated with elevated risk for BC overall (HR = 1.54, 95% CI 1.44–1.64), and risk was stronger in current users (HR = 1.67, 95% CI 1.56–1.79) and those with 10 or more years of use (Fig. 2). Significantly elevated risks for ever EPT use were seen for subtypes defined by ER/PR status (HR for ER+/PR+ = 1.67, 95% CI 1.51–1.85; for ER−/PR− = 1.57, 95% CI 1.24–1.99) (Fig. 2) and by histology (HR for invasive ductal carcinoma (IDC) = 1.53, 95% CI 1.40–1.67; for invasive lobular carcinoma (ILC), HR = 1.45, 95% CI 1.17–1.81; Fig. 3).

Duration of EPT use was linked to excess BC risk overall and to ductal and lobular cancer in situ (Additional file 2: Table S3), with stronger effects for lobular tumors (HR (DCIS) = 1.56, 95% CI 1.27–1.91; HR (LCIS) = 5.59, 95% CI 2.85–10.95). Similarly, excess risk associated with EPT duration was seen across all and hormone receptor subtypes (Additional file 2: Tables S6).

For ET users, no statistically significant associations were seen for in situ disease or invasive tumors defined by ER and/or PR status Additional file 2: Table S3), even among women in the lowest BMI group (Additional file 2: Table S4). In contrast, among EPT users, consistently elevated BC risk was observed for in situ and invasive breast cancer and across ER/PR subtypes (Additional file 2: Table S3). Risks did not vary significantly by BMI (Additional file 2: Table S6) but were highest in normal-weight women for BC overall and across histologic- and hormone receptor-defined cancers.

### Sub-cohort with follow-up questionnaire information

The effects of changes in MHT use during study follow-up on BC risk were assessed in the sub-cohort of women who completed the FUQ in 2004 (*n* = 75,398). Of these women, only 4.2% who reported never used MHT at baseline started use after study enrollment. Among women using ET or EPT at baseline, only 18.7% and 7.8%, respectively, continued to do so in the FUQ while 48.3% and 55%, respectively, had stopped, and the remainder (33% and 37%, respectively) either changed to other MHT formulations or did not provide adequate information on MHT use. Among those who discontinued MHT use, most did so after 2002 (63.4% of ET users and 48.7% of EPT), corresponding to the release of the initial WHI findings. We then excluded women who moved out of the catchment area prior to 2004 (*n* = 3540) and those diagnosed for BCs prior to 2004 (*n* = 3238), and the final dataset included 28,614 women with hysterectomy for ET analysis and 39,996 women with intact uteri for EPT analysis. Compared to never users, women who continued ET use had a significantly elevated BC risk (HR = 1.35, 95% CI 1.04–1.75), whereas those who had discontinued use were at a significantly lower risk (HR = 0.77, 95% CI 0.62–0.96) (Table 1). For EPT, the elevated BC risk for use at baseline increased in women continuing use through the 2004 questionnaire (HR = 1.80, 95% CI 1.39–2.32), but women who discontinued use during this period were no longer at significantly elevated risk.

**Table 1:** Breast cancer risk associated with estrogen-alone therapy (ET) in women with hysterectomy and estrogen plus progestin therapy (EPT) in women with intact uteri; restricted to postmenopausal women with follow-up MHT information in 2004 the National Institutes of Health-AARP Diet and Health Study cohort

	Women with hysterectomy (*n* = 28,614)		Women with intact uteri (*n* = 39,996)	
	*N* cases	HR (95%CIs)	*N* cases	HR (95%CIs)
**Total**	940		1539	
**MHT use**	**ET**		**EPT**	
Never	232	1.00 (referent)	436	1.00 (referent)
Ever	708	0.90 (0.74–1.11)	1103	**1.20 (1.06–1.37)**
**Ever MHT use**				
BMI < 25	420	0.88 (0.62–1.24)	695	**1.32 (1.09–1.61)**
BMI 25 < 30	284	1.10 (0.75–1.62)	479	1.10 (0.87–1.39)
BMI ≥ 30.0	214	0.74 (0.51–1.07)	338	1.21 (0.91–1.62)
		***p***_**trend**_ **= 0.87**		***p***_**trend**_ **< 0.0001**
**Recency**				
Former	596	**0.77 (0.62–0.96)**	1023	1.14 (0.99–1.30)
Current	112	**1.35 (1.04–1.75)**	80	**1.80 (1.39–2.32)**
**Duration, years**				
< 5	20	0.77 (0.39–1.50)	164	0.83 (0.64–1.08)
5–9	35	0.90 (0.55–1.48)	184	1.04 (0.81–1.32)
10–14	85	1.05 (0.73–1.51)	296	**1.37 (1.12–1.68)**
15–19	106	1.12 (0.80–1.56)	227	**1.39 (1.11–1.75)**
≥ 20	254	0.93 (0.72–1.21)	113	1.21 (0.86–1.72)
		***p***_**trend**_ **= 0.57**		***p***_**trend**_ **= 0.002**
**Time since last use, former users, years**				
< 5	193	0.69 (0.47–1.02)	358	1.23 (0.96–1.58)
5–9	80	0.82 (0.60–1.12)	189	1.18 (0.98–1.42)
≥ 10	78	0.80 (0.57–1.13)	92	1.18 (0.93–1.49)
		***p***_**trend**_ **< 0.0001**		***p***_**trend**_ **< 0.0001**

ET-alone analysis is restricted to women with hysterectomy. EPT analysis includes women with intact uterus

*MHT* hormone therapy; *p* values bold < 0.05

## Discussion

In this large prospective cohort with 15 years of follow-up, ET use at baseline was not associated with breast cancer risk overall or by histologic or hormone receptor-defined disease, but an elevated risk was suggested among women who continued ET use through the 2004 follow-up questionnaire (FUQ). In contrast, we found a consistent statistically significant elevated risk associated with EPT use both at baseline and in the sub-cohort with FUQ data for BC overall, invasive and in situ disease, and for histologic and hormone receptor subtypes, with significant dose-response effects across doses and EPT regimens. BC risk was highest (80% increased) in women who reported current EPT use on the 2004 FUQ compared to never users.

For ET, our largely null finding is consistent with long-term findings from the WHI [10, 11], but at odds with excess risk found in the preponderance of observational studies and the recently reported meta-analysis [1, 2, 7, 12–14]; in contrast, the elevated BC risk for EPT is in accord with a large body of evidence [2, 5, 7, 12, 15–17]. Reasons for discrepancies in the ET results may lie in differences in methodology and in the populations studied, particularly with respect to hysterectomy status [1, 5, 6, 13, 17]. Notably, few observational studies that found elevated risk for ET were restricted to women with hysterectomy [6, 13, 17], and indeed, unlike our findings, an earlier analysis of the NIH-AARP cohort found an elevated BC risk when all women were included in the comparison group [7]. Further discrepancies could be due to inadequate adjustment for effect modification by BMI, since MHT risks are more pronounced in thinner women [5]. We confirmed this effect in our EPT users, where compared to overweight or obese women, those of normal weight had higher BC risk overall, across histologic and ER/PR subtypes, and across categories of duration of MHT use.

With the accrual of more than 8300 BC cases, we were able to assess the associations between MHT and BC by subtypes defined by hormone receptor status and histology and for in situ cancer. We found no associations between ET and BC risk by hormone receptor status, which has been seen in some [18–20], but not most observational studies sufficiently powered to examine these associations [2, 13, 14, 21–24]. For EPT, we found a higher BC risk across all hormone receptor-defined subgroups, consistent with much of the evidence from observational and RCT reports [14, 16, 20–24].

We observed null associations between ET and all histologic subtypes of invasive BC. This has not been seen in the majority of observational studies, where excess risks across all histologic subtypes are reported [2, 25–27]. For EPT use, the elevated invasive BC risk across histologic subtypes is consistent with evidence from observational studies and the WHI [2, 16, 19, 22, 25–27]; however, unlike our study, most suggest a stronger association with lobular versus ductal cancer.

Few studies have assessed MHT risk for in situ BC. In our cohort, no significantly elevated risks for in situ disease were seen for ET use, but excess risk of both DCIS and LCIS was seen among EPT users, with stronger effects for in situ lobular cancers. For DCIS, the lack of association with ET [23, 27] alongside an elevated risk for EPT use [23, 27, 28] has been seen. Evidence for a role of ET or EPT on risk of LCIS is lacking in the literature.

For EPT users, BC risk was similarly elevated for sequential and continuous regimens, consistent with most studies [5, 12, 27, 29], and risks were of similar magnitude across progestin doses. For women with updated FUQ MHT information, those with 10 or more years of EPT use were at an approximate 40% increased risk, which is lower than the doubling in risk from observational studies that only reflect usage at the time of enrollment. Although incomplete, our estimates of MHT effects incorporated changes in usage patterns over approximately half the follow-up period and thus may be better estimates of long-term BC risk.

This study has several strengths, including its prospective design, large size, and extensive information on hormone therapy and potential confounders both at enrollment and several years after the study began. The large number of cases allowed us to analyze breast cancer risk for histology and hormone receptor subtype, which has been inadequately addressed in the literature, and to consider the association between MHT and BC risk in women with and without hysterectomies. Our risk estimates account for changes in usage patterns from baseline through 2004, which is significant in that approximately 50% of women using MHT at enrollment no longer did so after safety concerns about EPT were published, consistent with the decline in MHT prescriptions subsequent to the release of WHI findings in 2002 [30]. Women who continued to use EPT at the time of the 2004 FUQ had the highest BC risk. Although MHT treatment was not validated for NIH-AARP, in order to permit a comparison to the WHI, we limited ET only users to those reporting use of Premarin (CEE), and for EPT users, to those reporting formulations using CEE and medroxyprogesterone acetate (MPA). Another limitation of this study is that while the 2004 FUQ provided valuable updates to MHT usage patterns, we lacked information for the latter part of the 15-year follow-up period and could not account for changes in MHT use from 2005 to the end of the 2011 follow-up period. While it is improbable that never MHT users in 2004 would later start treatment, a more likely scenario is that some women using hormone therapy in 2004 later discontinued use. A somewhat more severe limitation is that only 58% of eligible women in the full cohort satisfactorily completed the follow-up survey in 2004. Nonetheless, in light of changes in MHT use captured in the 2004 FUQ, we speculate that risk estimates from the full cohort using only baseline MHT use may be biased.

## Conclusions

In summary, EPT users had a sustained increased risk for in situ and invasive BC overall and across subtypes by histology and hormone receptor status, even after cessation.

## Supplementary Information

**Additional file 1: Figure S1.** Flow chart of inclusion criteria and study population.

**Additional file 2: Table S1.** Distribution of participants and breast cancer cases in different race subgroups. **Table S2.** Selected risk factors for hormone therapy use among eligible postmenopausal women in the entire National Institutes of Health-AARP Diet and Health Study cohort in 1996–1997. **Table S3.** Hormone therapy at baseline and risk of in situ breast cancer and invasive breast cancer by estrogen and progesterone receptor status, among postmenopausal women in the National Institutes of Health-AARP Diet and Health Study cohort (1996/1997–2011). **Table S4.** Associations of estrogen alone therapy (ET) at baseline with risk of breast cancer, by body mass index (BMI) among postmenopausal women with hysterectomy, the National Institutes of Health-AARP Diet and Health Study cohort (1996/1997–2011). **Table S5.** Associations of estrogen alone therapy (ET) at baseline with risk of invasive ductal and lobular breast cancer by hormone receptor status, postmenopausal women with hysterectomy, the National Institutes of Health-AARP Diet and Health Study cohort (1996/1997–2011). **Table S6.** Associations of estrogen plus progestin therapy (EPT) use at baseline with risk of breast cancer, by body mass index (BMI), Postmenopausal women with intact uteri, the National Institutes of Health-AARP Diet and Health Study cohort (1996/1997–2011). **Table S7.** Associations of estrogen plus progestin therapy (EPT) use at baseline with risk of invasive ductal and lobular breast cancer by hormone receptor status, postmenopausal women with intact uteri, the National Institutes of Health-AARP Diet and Health Study cohort (1996/ 1997–2011).

## Data Availability

The dataset for this study is available from the corresponding author on reasonable request.

## Abbreviations

BC: Breast cancer
MHT: Menopausal hormone therapy
ET: Estrone-alone therapy
CEE: Combined equine estrogens
EPT: Estrogen plus progestin therapy
ER: Estrogen receptor
PR: Progesterone receptor
IDC: Invasive ductal carcinoma
ILC: Invasive lobular carcinoma
DCIS: Ductal carcinoma in situ
LCIS: Lobular carcinoma in situ
NIH-AARP cohort: National Institutes of Health American Association of Retired Persons cohort
HR: Hazard ratio
CI: Confidence interval
