# Supplementary material for: Use of postmenopausal hormone therapies and risk of histology- and hormone receptor-defined breast cancer: results from a 15-year prospective analysis of NIH-AARP cohort
Source: Breast Cancer Res. 2020 Nov 25;22:129. doi: 10.1186/s13058-020-01365-9 (PMC7687781; doi:10.1186/s13058-020-01365-9)
Supplement: Supplementary file 1 — Additional file 1: Figure S1. Flow chart of inclusion criteria and study population. [file 13058_2020_1365_MOESM1_ESM.pdf]

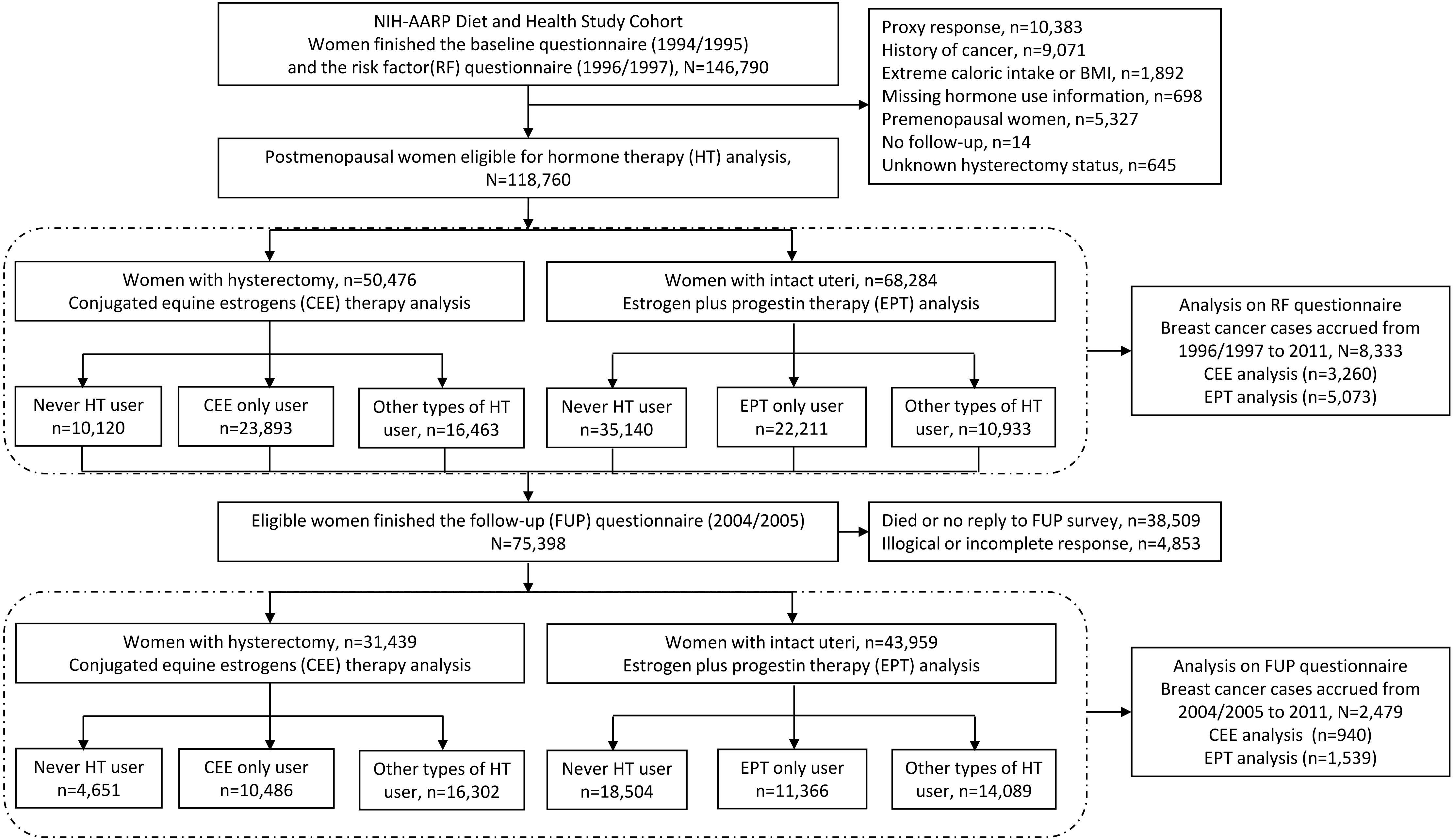
NIH-AARP Diet and Health Study Cohort
Women finished the baseline questionnaire (1994/1995)
and the risk factor(RF) questionnaire (1996/1997), N=146,790
Proxy response, n=10,383
History of cancer, n=9,071
Extreme caloric intake or BMI, n=1,892
Missing hormone use information, n=698
Premenopausal women, n=5,327
No follow-up, n=14
Unknown hysterectomy status, n=645
Postmenopausal women eligible for hormone therapy (HT) analysis, N=118,760
Women with hysterectomy, n=50,476
Conjugated equine estrogens (CEE) therapy analysis
Women with intact uteri, n=68,284
Estrogen plus progestin therapy (EPT) analysis
Never HT user n=10,120
CEE only user n=23,893
Other types of HT user, n=16,463
Never HT user n=35,140
EPT only user n=22,211
Other types of HT user, n=10,933
Analysis on RF questionnaire
Breast cancer cases accrued from 1996/1997 to 2011, N=8,333
CEE analysis (n=3,260)
EPT analysis (n=5,073)
Eligible women finished the follow-up (FUP) questionnaire (2004/2005)
N=75,398
Died or no reply to FUP survey, n=38,509
Illogical or incomplete response, n=4,853
Women with hysterectomy, n=31,439
Conjugated equine estrogens (CEE) therapy analysis
Women with intact uteri, n=43,959
Estrogen plus progestin therapy (EPT) analysis
Never HT user n=4,651
CEE only user n=10,486
Other types of HT user, n=16,302
Never HT user n=18,504
EPT only user n=11,366
Other types of HT user, n=14,089
Analysis on FUP questionnaire
Breast cancer cases accrued from 2004/2005 to 2011, N=2,479
CEE analysis (n=940)
EPT analysis (n=1,539)
