# Supplementary material for: Use of postmenopausal hormone therapies and risk of histology- and hormone receptor-defined breast cancer: results from a 15-year prospective analysis of NIH-AARP cohort
Source: Breast Cancer Res. 2020 Nov 25;22:129. doi: 10.1186/s13058-020-01365-9 (PMC7687781; doi:10.1186/s13058-020-01365-9)
Supplement: Supplementary file 2 — Additional file 2: Table S1. Distribution of participants and breast cancer cases in different race subgroups. Table S2. Selected risk factors for hormone therapy use among eligible postmenopausal women in the entire National Institutes of Health-AARP Diet and Health Study cohort in 1996–1997. Table S3. Hormone therapy at baseline and risk of in situ breast cancer and invasive breast cancer by estrogen and progesterone receptor status, among postmenopausal women in the National Institutes of Health-AARP Diet and Health Study cohort (1996/1997–2011). Table S4. Associations of estrogen alone therapy (ET) at baseline with risk of breast cancer, by body mass index (BMI) among postmenopausal women with hysterectomy, the National Institutes of Health-AARP Diet and Health Study cohort (1996/1997–2011). Table S5. Associations of estrogen alone therapy (ET) at baseline with risk of invasive ductal and lobular breast cancer by hormone receptor status, postmenopausal women with hysterectomy, the National Institutes of Health-AARP Diet and Health Study cohort (1996/1997–2011). Table S6. Associations of estrogen plus progestin therapy (EPT) use at baseline with risk of breast cancer, by body mass index (BMI), Postmenopausal women with intact uteri, the National Institutes of Health-AARP Diet and Health Study cohort (1996/1997–2011). Table S7. Associations of estrogen plus progestin therapy (EPT) use at baseline with risk of invasive ductal and lobular breast cancer by hormone receptor status, postmenopausal women with intact uteri, the National Institutes of Health-AARP Diet and Health Study cohort (1996/ 1997–2011). [file 13058_2020_1365_MOESM2_ESM.docx]

**Table S1**

| **Analysis group** | **Group** | **Race / Ethnicity** | | | | | | | | |
| --- | --- | --- | --- | --- | --- | --- | --- | --- | --- | --- |
|  |  | **Non-Hispanic White** | **Non-Hispanic Black** | **Hispanic** | **Asian** | **Pacific Islander** | **American Indian/ Alaskan Native** | **Unknown** | **Total** |  |
| **ET alone analysis** | **BC cases** | 2948 | 189 | 40 | 28 | 1 | 8 | 46 | 3260 |  |
|  | **Total** | 45496 | 2962 | 851 | 345 | 40 | 169 | 613 | 50476 |  |
| **EPT**  **analysis** | **BC cases** | 4739 | 157 | 53 | 58 | 7 | 9 | 50 | 5073 |  |
|  | **Total** | 62951 | 2569 | 1054 | 768 | 64 | 149 | 729 | 68284 |  |
| **Total**  **dataset** | **BC cases** | 7687 | 346 | 93 | 86 | 8 | 17 | 96 | 8333 |  |
|  | **Total** | 108447 | 5531 | 1905 | 1113 | 104 | 318 | 1342 | 118760 |  |

**Table S2**

|  | **Estrogen (ET) therapy**  **(n=50,476)** | | | |  | **Estrogen plus progestin (EPT) therapy (n=68,284)** | | | |  | |
| --- | --- | --- | --- | --- | --- | --- | --- | --- | --- | --- | --- |
| **Characteristic** | **Never HT** | | **ET** | **Other Preparations** | | **Never HT** | **ET** | | **Other Preparations** | |  |
|  | **n (%)** | **n (%)** | | **n (%)** | | **n (%)** | **n (%)** | | **n (%)** | |  |
| **N** | 10120 (20.1) | 23893 (47.3) | | 16463 (32.6) | | 35140 (51.5) | 22211 (32.5) | | 10933 (16.0) | |  |
| **N breast cancer cases** | 615 | 1489 | | 1156 | | 2170 | 2110 | | 793 | |  |
| **Person-years** | 128496.99 | 309048.96 | | 212896.79 | | 451619.34 | 288006.38 | | 139536.47 | |  |
| **Age at entry, years** |  |  | |  | |  |  | |  | |  |
| <57 | 1214 (12.0) | 3925 (16.4) | | 3317 (20.2) | | 4514 (12.9) | 4833 (21.8) | | 1457 (13.3) | |  |
| 57-60 | 1500 (14.8) | 4649 (19.5) | | 3599 (21.9) | | 5706 (16.2) | 5935 (26.7) | | 2077 (19.0) | |  |
| 61-64 | 2455 (24.3) | 5745 (24.0) | | 3975 (24.2) | | 8168 (23.2) | 5343 (24.1) | | 2475 (22.6) | |  |
| 65-68 | 3199 (31.6) | 6338 (26.5) | | 3789 (23.0) | | 10911 (31.1) | 4391 (19.8) | | 3076 (28.1) | |  |
| ≥69 | 1752 (17.3) | 3236 (13.5) | | 1783 (10.8) | | 5841 (16.6) | 1709 (7.7) | | 1848 (16.9) | |  |
| **Race** |  |  | |  | |  |  | |  | |  |
| White | 8579 (84.8) | 21891 (91.6) | | 15026 (91.3) | | 31953 (90.9) | 20998 (94.5) | | 10000 (91.5) | |  |
| Nonwhite | 1541 (15.2) | 2002 (8.4) | | 1437 (8.7) | | 3187 (9.1) | 1213 (5.5) | | 933 (8.5) | |  |
| **Age at first birth, years** |  |  | |  | |  |  | |  | |  |
| nulliparous | 1072 (10.6) | 2643 (11.1) | | 1935 (11.8) | | 6081 (17.3) | 3679 (16.6) | | 1640 (15.0) | |  |
| <20 | 2252 (22.3) | 5392 (22.6) | | 3402 (20.7) | | 4484 (12.8) | 2621 (11.8) | | 1586 (14.5) | |  |
| 20-24 | 4799 (47.4) | 11074 (46.4) | | 7627 (46.3) | | 14763 (42.0) | 9345 (42.1) | | 4720 (43.2) | |  |
| 25-29 | 1525 (15.1) | 3683 (15.4) | | 2640 (16.0) | | 6902 (19.6) | 4732 (21.3) | | 2113 (19.3) | |  |
| ≥30 | 390 (3.9) | 937 (3.9) | | 696 (4.2) | | 2587 (7.4) | 1641 (7.4) | | 762 (7.0) | |  |
| Unknown | 82 (0.8) | 164 (0.7) | | 163 (1.0) | | 323 (0.9) | 193 (0.9) | | 112 (1.0) | |  |
| **Age at menopause, years** |  |  | |  | |  |  | |  | |  |
| <45 | 162 (1.6) | 268 (1.1) | | 861 (5.2) | | 4263 (12.1) | 1778 (8.0) | | 4721 (43.2) | |  |
| 45-49 | 280 (2.8) | 428 (1.8) | | 257 (1.6) | | 9714 (27.6) | 5258 (23.7) | | 1329 (12.2) | |  |
| 50-54 | 399 (3.9) | 615 (2.6) | | 527 (3.2) | | 17035 (48.5) | 10635 (47.9) | | 2923 (26.7) | |  |
| ≥55 | 74 (0.7) | 106 (0.4) | | 167 (1.0) | | 3409 (9.7) | 2777 (12.5) | | 1126 (10.3) | |  |
| Surgical | 9090 (89.8) | 22289 (93.3) | | 14481 (88.0) | | 445 (1.3) | 181 (0.8) | | 374 (3.4) | |  |
| Unknown | 115 (1.2) | 187 (0.8) | | 170 (1.0) | | 274 (0.8) | 1582 (7.1) | | 460 (4.2) | |  |
| **Family history of breast cancer** |  | |  |  | |  |  |  |  |  |  |
| No | 6650 (65.7) | 16359 (68.5) | | 11216 (68.1) | | 23540 (67.0) | 16040 (72.2) | | 7409 (67.8) | |  |
| Yes | 1520 (15.0) | 3168 (13.3) | | 2335 (14.2) | | 4842 (13.8) | 2799 (12.6) | | 1396 (12.8) | |  |
| Unknown | 1950 (19.3) | 4366 (18.3) | | 2912 (17.7) | | 6758 (19.2) | 3372 (15.2) | | 2128 (19.5) | |  |
| **Breast biopsy** |  |  | |  | |  |  | |  | |  |
| None | 7520 (74.3) | 17285 (72.3) | | 11550 (70.2) | | 28452 (81.00) | 16582 (74.7) | | 8346 (76.3) | |  |
| 1 | 1561 (15.4) | 4193 (17.6) | | 3035 (18.4) | | 4517 (12.9) | 3880 (17.5) | | 1731 (15.8) | |  |
| 2 | 482 (4.8) | 1260 (5.3) | | 944 (5.7) | | 1159 (3.3) | 1002 (4.5) | | 462 (4.2) | |  |
| ≥3 | 505 (5.0) | 1081 (4.5) | | 871 (5.3) | | 913 (2.6) | 708 (3.2) | | 355 (3.2) | |  |
| Unknown | 52 (0.51) | 74 (0.3) | | 63 (0.4) | | 99 (0.28) | 39 (0.2) | | 39 (0.4) | |  |
| **Alcohol consumption, drink/day** |  | | |  | |  |  | |  | |  |
| Never | 3883 (38.4) | 6900 (28.9) | | 4691 (28.5) | | 10708 (30.5) | 4649 (20.9) | | 2888 (26.4) | |  |
| ≤1 | 5251 (51.9) | 14050 (58.8) | | 9605 (58.3) | | 19749 (56.2) | 13922 (62.7) | | 6414 (58.7) | |  |
| 1-3 | 755 (7.5) | 2379 (10.0) | | 1742 (10.6) | | 3633 (10.3) | 3011 (13.6) | | 1283 (11.7) | |  |
| ≥3 | 231 (2.3) | 564 (2.4) | | 425 (2.6) | | 1050 (3.0) | 629 (2.8) | | 348 (3.2) | |  |
| **Body mass index ( BMI, kg/m^2^)** |  | | |  | |  |  | |  | |  |
| <25 | 3146 (31.1) | 10401 (43.5) | | 7409 (45.0) | | 14538 (41.4) | 12098 (54.5) | | 5139 (47.0) | |  |
| 25.0-<30 | 3352 (33.1) | 7939 (33.2) | | 5365 (32.6) | | 11168 (31.8) | 6443 (29.0) | | 3443 (31.5) | |  |
| ≥30.0 | 3269 (32.3) | 4972 (20.8) | | 3275 (19.9) | | 8331 (23.7) | 3221 (14.5) | | 2046 (18.7) | |  |
| Unknown | 353 (3.5) | 581 (2.4) | | 414 (2.5) | | 1103 (3.1) | 449 (2.0) | | 305 (2.8) | |  |
| **Physical activity** |  |  | |  | |  |  | |  | |  |
| Never or rarely | 2868 (28.7) | 4889 (20.6) | | 3309 (20.3) | | 8268 (23.8) | 3533 (16.0) | | 2213 (20.5) | |  |
| 1-3 times/ month | 1386 (13.9) | 3422 (14.4) | | 2316 (14.2) | | 4864 (14.0) | 3169 (14.3) | | 1475 (13.6) | |  |
| 1-2 times/ week | 2038 (20.4) | 5098 (21.5) | | 3430 (21.0) | | 7344 (21.1) | 4787 (21.7) | | 2250 (20.8) | |  |
| 3-4 times/ week | 2211 (22.1) | 6321 (26.7) | | 4475 (27.4) | | 8372 (24.1) | 6474 (29.3) | | 2919 (27.0) | |  |
| ≥5 times/ week | 1503 (15.0) | 3992 (16.8) | | 2777 (17.0) | | 5948 (17.1) | 4133 (18.7) | | 1963 (18.1) | |  |
| **Number mammograms** |  |  | |  | |  |  | |  | |  |
| ≤1 | 4607 (45.5) | 5467 (22.9) | | 3452 (21.0) | | 16210 (46.1) | 3291 (14.8) | | 3005 (27.5) | |  |
| ≥2 | 5395 (53.3) | 18344 (76.8) | | 12882 (78.3) | | 18640 (53.0) | 18865 (84.9) | | 7815 (71.5) | |  |
| Unknown | 118 (1.2) | 82 (0.3) | | 129 (0.8) | | 290 (0.8) | 55 (0.3) | | 113 (1.0) | |  |

**Table S3**

| **Hormone therapy** | **Variable** | **In situ breast cancer, HRs (95%CI)** | | **Invasive breast cancer by hormone receptor status, HR (95%CIs)** | | | |
| --- | --- | --- | --- | --- | --- | --- | --- |
|  |  | **DCIS** | **LCIS** | **ER+** | **ER-** | **PR+** | **PR-** |
| **Estrogen alone therapy (ET), women with hysterectomy (n=50,476)** | **No. cases** | 375 | 40 | 1455 | 304 | 1189 | 537 |
|  | **Never MHT** | 1.00 referent | 1.00 referent | 1.00 referent | 1.00 referent | 1.00 referent | 1.00 referent |
|  | **Ever ET use** | 1.08 (0.80-1.45) | 1.59 (0.52-4.85) | 1.04 (0.90-1.20) | 1.01 (0.74-1.38) | 0.97 (0.83-1.14) | 1.10 (0.87-1.40) |
|  | **Recency** | |  |  |  |  |  |
|  | Former | 1.00 (0.65-1.55) | 1.12 (0.20-6.16) | 0.97 (0.79-1.21) | 0.99 (0.63-1.56) | 0.86 (0.68-1.10) | 1.16 (0.83-1.63) |
|  | Current | 1.11 (0.82-1.50) | 1.71 (0.55-5.33) | 1.05 (0.90-1.22) | 1.03 (0.74-1.42) | 1.01 (0.85-1.19) | 1.08 (0.84-1.39) |
|  | **Duration current users, years** | | | |  |  |  |
|  | <5 | 1.06 (0.64-1.77) | - | 0.93 (0.72-1.22) | 1.04 (0.62-1.77) | 0.93 (0.70-1.24) | 0.97 (0.64-1.47) |
|  | 5-9 | 1.10 (0.67-1.79) | 2.74 (0.70-10.72) | 1.15 (0.91-1.45) | 1.06 (0.63-1.76) | 1.12 (0.86-1.45) | 1.14 (0.77-1.67) |
|  | ≥10 | 1.13 (0.82-1.57) | 1.92 (0.58-6.34) | 1.07 (0.91-1.26) | 0.98 (0.69-1.40) | 1.00 (0.84-1.20) | 1.10(0.84-1.44) |
|  | ***P*_trend_** | 0.38 | 0.19 | 0.33 | 0.95 | 0.84 | 0.47 |
| **Estrogen plus progestin therapy (EPT), women with intact uteri (n=68,284)** | **No. of cases** | 523 | 75 | 2474 | 438 | 2058 | 773 |
|  | **Never MHT** | 1.00 referent | 1.00 referent | 1.00 referent | 1.00 referent | 1.00 referent | 1.00 referent |
|  | **Ever EPT use** | **1.56 (1.27-1.91)** | **5.59 (2.85-10.95)** | **1.68 (1.53-1.85)** | **1.55 (1.24-1.95)** | **1.67 (1.51-1.85)** | **1.56 (1.32-1.85)** |
|  | **Recency** | |  |  |  |  |  |
|  | Former | 1.05 (0.70-1.56) | **3.23 (1.12-9.31)** | 1.12 (0.93-1.34) | 1.38 (0.94-2.05) | 1.14 (0.94-1.39) | 1.19 (0.87-1.63) |
|  | Current | **1.67 (1.35-2.06)** | **6.15 (3.09-12.24)** | **1.85 (1.68-2.04)** | **1.58 (1.24-2.01)** | **1.83 (1.65-2.04)** | **1.65 (1.38-1.97)** |
|  | **Duration current users, years** | | | |  |  |  |
|  | <5 | **1.44 (1.07-1.95)** | **3.61 (1.45-8.95)** | **1.46 (1.27-1.69)** | 1.36 (0.96-1.93) | **1.40 (1.19-1.64)** | **1.46 (1.13-1.88)** |
|  | 5-9 | **1.93 (1.47-2.54)** | **8.27 (3.82-17.89)** | **1.79 (1.57-2.05)** | **1.79 (1.30-2.46)** | **1.75 (1.51-2.03)** | **1.80 (1.42-2.29)** |
|  | ≥10 | **1.64 (1.21-2.21)** | **7.09 (3.12-16.12)** | **2.34 (2.06-2.67)** | **1.64 (1.15-2.33)** | **2.42 (2.10-2.78)** | **1.72 (1.33-2.23)** |
|  | ***P*_trend_** | **<0.0001** | **<0.0001** | **<0.0001** | **0.0003** | **<0.0001** | **<0.0001** |

DCIS, ductal carcinoma in situ; LCIS, lobular carcinoma in situ; ER, estrogen receptor; PR, progesterone receptor; bold indicates p value< 0.05

**Table S4**

| **Hormone therapy** | **Total breast cancer** | | **Invasive breast cancer by histological type or by hormone receptor status (HR, 95%CIs)** | | | | | | |
| --- | --- | --- | --- | --- | --- | --- | --- | --- | --- |
|  |  |  | **IDC** | **ILC** | **Mixed** | **Other** | **ER+PR+** | **ER+PR-** | **ER-PR-** |
| **No. of cases** | 3260 | | 1811 | 283 | 201 | 530 | 740 | 178 | 178 |
| **Never MHT** | 1.00 referent | | 1.00 referent | 1.00 referent | 1.00 referent | 1.00 referent | 1.00 referent | 1.00 referent | 1.00 referent |
| **Ever Baseline ET use** | | |  |  |  |  |  |  |  |
| BMI <25 | 1.12 (0.95-1.33) | | 1.03 (0.83-1.30) | 1.24 (0.70-2.20) | 1.82 (0.85-3.90) | 1.14 (0.74-1.76) | 1.22 (0.91-1.63) | 1.40 (0.72-2.72) | 0.71 (0.42-1.17) |
| BMI 25-<30 | 1.03 (0.87-1.23) | | 0.94 (0.75-1.17) | 0.95 (0.54-1.69) | 1.05 (0.54-2.03) | 1.56 (0.99-2.46) | 0.83 (0.63-1.10) | 1.20 (0.68-2.10) | 1.42 (0.75-2.69) |
| BMI ≥30.0 | 0.92 (0.77-1.10) | | 0.95 (0.76-1.20) | 0.84 (0.46-1.56) | 0.66 (0.29-1.50) | 0.89 (0.58-1.36) | 0.96 (0.72-1.27) | 1.12 (0.56-2.25) | 1.07 (0.58-1.98) |
| **Former user** |  | |  |  |  |  |  |  |  |
| BMI <25 | 0.83 (0.64-1.09) | | 0.76 (0.53-1.09) | 0.86 (0.35-2.12) | 0.48 (0.10-2.28) | 1.12 (0.60-2.08) | 0.79 (0.49-1.28) | 1.53 (0.63-3.71) | **0.34 (0.12-0.98)** |
| BMI 25-<30 | 1.00 (0.78-1.27) | | 0.88 (0.64-1.22) | 0.94 (0.41-2.17) | 0.98 (0.37-2.60) | 1.74 (0.96-3.15) | 0.89 (0.6-1.34) | 0.99 (0.43-2.29) | 1.33 (0.55-3.21) |
| BMI ≥30.0 | 0.98 (0.76-1.26) | | 0.95 (0.68-1.33) | 1.20 (0.52-2.75) | 0.89 (0.28-2.82) | 0.93 (0.51-1.71) | 0.93 (0.61-1.41) | 1.95 (0.85-4.48) | 1.22 (0.52-2.82) |
| **Current user** | |  |  |  |  |  |  |  |  |
| BMI <25 | 1.18 (0.99-1.41) | | 1.08 (0.86-1.36) | 1.33 (0.74-2.39) | 2.14 (0.99-4.62) | 1.14 (0.73-1.78) | 1.31 (0.97-1.77) | 1.31 (0.66-2.59) | 0.79 (0.47-1.33) |
| BMI 25-<30 | 1.05 (0.88-1.26) | | 0.96 (0.77-1.21) | 0.97 (0.53-1.76) | 1.08 (0.54-2.14) | 1.49 (0.93-2.40) | 0.82 (0.61-1.10) | 1.28 (0.71-2.29) | 1.46 (0.75-2.83) |
| BMI ≥30.0 | 0.90 (0.74-1.09) | | 0.95 (0.74-1.21) | 0.70 (0.35-1.38) | 0.52 (0.21-1.33) | 0.89 (0.56-1.41) | 0.95 (0.71-1.29) | 0.85 (0.39-1.84) | 1.03 (0.53-1.99) |
| **Duration of use, years** | | |  |  |  |  |  |  |  |
| **<5** |  | |  |  |  |  |  |  |  |
| BMI <25 | 0.96 (0.75-1.22) | | 0.86 (0.62-1.19) | 1.22 (0.56-2.62) | 1.26 (0.43-3.68) | 0.92 (0.49-1.73) | 0.98 (0.64-1.50) | 1.65 (0.72-3.77) | 0.56 (0.26-1.23) |
| BMI 25-<30 | 1.05 (0.83-1.32) | | 1.02 (0.76-1.37) | 0.96 (0.44-2.09) | 0.83 (0.31-2.21) | 1.66 (0.93-2.96) | 0.85 (0.58-1.26) | 1.00 (0.44-2.25) | 1.51 (0.67-3.41) |
| BMI ≥30.0 | 0.92 (0.72-1.18) | | 1.00 (0.73-1.37) | 0.84 (0.35-2.02) | 1.10 (0.40-3.02) | 0.72 (0.38-1.39) | 0.96 (0.65-1.42) | 1.78 (0.78-4.03) | 1.13 (0.50-2.57) |
| **5-9** |  | |  |  |  |  |  |  |  |
| BMI <25 | 1.11 (0.87-1.41) | | 1.01 (0.73-1.40) | 1.27 (0.57-2.82) | 2.31 (0.89-5.98) | 0.72 (0.35-1.47) | 1.29 (0.85-1.94) | 1.48 (0.62-3.55) | 0.49 (0.21-1.16) |
| BMI 25-<30 | 1.14 (0.88-1.48) | | 0.97 (0.69-1.37) | 1.93 (0.94-3.98) | 0.61 (0.17-2.17) | 1.79 (0.95-3.41) | 0.95 (0.61-1.46) | 1.58 (0.71-3.51) | 1.40 (0.55-3.57) |
| BMI ≥30.0 | 0.95 (0.7-1.28) | | 0.96 (0.65-1.41) | 0.96 (0.35-2.61) | 0.89 (0.24-3.29) | 0.96 (0.46-2.00) | 1.15 (0.74-1.79) | 0.45 (0.10-2.02) | 0.99 (0.36-2.74) |
| **≥10** |  | |  |  |  |  |  |  |  |
| BMI <25 | 1.19 (1.00-1.43) | | 1.11 (0.87-1.41) | 1.27 (0.69-2.32) | 1.90 (0.86-4.18) | 1.32 (0.84-2.09) | 1.30 (0.95-1.77) | 1.32 (0.66-2.67) | 0.84 (0.49-1.45) |
| BMI 25-<30 | 1.00 (0.83-1.20) | | 0.89 (0.69-1.13) | 0.67 (0.34-1.35) | 1.28 (0.63-2.57) | 1.42 (0.86-2.33) | 0.79 (0.58-1.08) | 1.15 (0.62-2.14) | 1.34 (0.67-2.70) |
| BMI ≥30.0 | 0.92 (0.75-1.13) | | 0.93 (0.71-1.21) | 0.83 (0.40-1.71) | 0.38 (0.12-1.20) | 0.92 (0.56-1.51) | 0.90 (0.64-1.25) | 0.94 (0.40-2.19) | 1.09 (0.53-2.22) |

MHT, menopausal hormone therapy; IDC, invasive ductal carcinoma; ILC, invasive lobular carcinoma; ER, estrogen receptor; PR, progesterone receptor Bold indicates p value< 0.05.

**Table S5**

| **Hormone therapy** | **Breast cancer subtype by hormone receptor status**  **(HR, 95%CIs)** | | |
| --- | --- | --- | --- |
|  | **ER+PR+** | **ER+PR-** | **ER-PR-** |
| **No. cases** |  |  |  |
| Total breast cancer | 1158 | 258 | 277 |
| Invasive ductal cancer | 809 | 182 | 225 |
| Invasive lobular cancer | 150 | 37 | 7 |
| **Never MHT** | 1.00 referent | 1.00 referent | 1.00 referent |
| **Ever ET use** |  |  |  |
| Total breast cancer | 0.97 (0.82-1.13) | 1.21 (0.85-1.73) | 1.00 (0.72-1.39) |
| Invasive ductal cancer | 0.94 (0.78-1.14) | 1.37 (0.88-2.11) | 1.09 (0.75-1.57) |
| Invasive lobular cancer | 0.91 (0.58-1.41) | 1.13 (0.46-2.79) | 0.42 (0.06-3.09) |
| **Former user** |  |  |  |
| Total breast cancer | 0.85 (0.67-1.09) | 1.40 (0.87-2.25) | 0.98 (0.60-1.58) |
| Invasive ductal cancer | 0.84 (0.63-1.12) | 1.47 (0.82-2.64) | 1.00 (0.58-1.72) |
| Invasive lobular cancer | 0.72 (0.35-1.47) | 1.94 (0.65-5.83) | - |
| **Current user** |  |  |  |
| Total breast cancer | 1.00 (0.85-1.18) | 1.14 (0.79-1.65) | 1.02 (0.72-1.43) |
| Invasive ductal cancer | 0.97 (0.80-1.19) | 1.30 (0.83-2.05) | 1.12 (0.77-1.64) |
| Invasive lobular cancer | 0.94 (0.60-1.49) | 0.93 (0.36-2.43) | 0.53 (0.07-4.01) |
| **Duration of use, years** |  |  |  |
| **<5** |  |  |  |
| Total breast cancer | 0.90 (0.71-1.13) | 1.35 (0.86-2.14) | 1.03 (0.66-1.60) |
| Invasive ductal cancer | 0.86 (0.66-1.14) | 1.47 (0.84-2.58) | 1.16 (0.72-1.88) |
| Invasive lobular cancer | 0.63 (0.31-1.26) | 1.73 (0.60-5.05) | - |
| **5-9** |  |  |  |
| Total breast cancer | 1.09 (0.85-1.38) | 1.20 (0.71-2.03) | 0.90 (0.54-1.51) |
| Invasive ductal cancer | 1.04 (0.77-1.39) | 1.53 (0.83-2.81) | 0.95 (0.54-1.70) |
| Invasive lobular cancer | 1.17 (0.63-2.19) | 0.96 (0.24-3.84) | 1.48 (0.12-17.98) |
| **≥10** |  |  |  |
| Total breast cancer | 0.97 (0.82-1.16) | 1.14 (0.77-1.69) | 1.03 (0.72-1.48) |
| Invasive ductal cancer | 0.96 (0.78-1.18) | 1.24 (0.77-2.01) | 1.12 (0.75-1.68) |
| Invasive lobular cancer | 0.96 (0.59-1.56) | 0.94 (0.34-2.62) | 0.33 (0.03-3.80) |

ER, estrogen receptor; PR, progesterone receptor

Bold indicates p value< 0.05.

**Table S6**

| **Hormone therapy** | **Total breast cancer** | **Invasive breast cancer by histological type or by hormone receptor status (HR, 95%CIs)** | | | | | | |
| --- | --- | --- | --- | --- | --- | --- | --- | --- |
|  |  | **IDC** | **ILC** | **Mixed** | **Other** | **ER+PR+** | **ER+PR-** | **ER-PR-** |
| **No. of cases** | 5073 | 2919 | 468 | 367 | 692 | 1473 | 287 | 289 |
| **Never MHT** | 1.00 referent | 1.00 referent | 1.00 referent | 1.00 referent | 1.00 referent | 1.00 referent | 1.00 referent | 1.00 referent |
| **Ever EPT use** |  |  |  |  |  |  |  |  |
| BMI <25 | **1.77 (1.60-1.95)** | **1.76 (1.55-2.01)** | **1.74 (1.26-2.40)** | **2.14 (1.46-3.13)** | **1.59 (1.21-2.10)** | **1.95 (1.66-2.29)** | **1.72 (1.22-2.42)** | **1.65 (1.17-2.34)** |
| BMI 25-<30 | **1.37 (1.22-1.54)** | **1.35 (1.15-1.58)** | **1.55 (1.05-2.29)** | **2.07 (1.38-3.09)** | 1.03 (0.75-1.42) | **1.64 (1.36-1.96)** | 1.36 (0.85-2.19) | 1.51 (0.98-2.31) |
| BMI ≥30.0 | **1.36 (1.18-1.57)** | **1.37 (1.14-1.66)** | 0.85 (0.48-1.48) | **2.35 (1.32-4.17)** | 1.13 (0.76-1.68) | **1.39 (1.11-1.74)** | 1.15 (0.61-2.16) | 1.52 (0.90-2.59) |
| **Former user** |  |  |  |  |  |  |  |  |
| BMI <25 | 1.10 (0.91-1.34) | 1.19 (0.93-1.53) | 0.96 (0.49-1.87) | 1.14 (0.54-2.43) | 0.88 (0.49-1.58) | 1.28 (0.95-1.73) | 1.16 (0.61-2.21) | 1.21 (0.63-2.30) |
| BMI 25-<30 | 1.10 (0.88-1.36) | 0.96 (0.70-1.31) | 1.53 (0.80-2.93) | 1.53 (0.74-3.14) | 0.88 (0.48-1.60) | 1.13 (0.79-1.60) | 0.56 (0.17-1.81) | **2.07 (1.12-3.83)** |
| BMI ≥30.0 | 0.95 (0.72-1.26) | 0.93 (0.65-1.35) | 0.57 (0.18-1.82) | 1.20 (0.36-3.98) | 1.04 (0.52-2.07) | 1.09 (0.73-1.64) | 0.58 (0.14-2.40) | 0.99 (0.35-2.79) |
| **Current user** |  |  |  |  |  |  |  |  |
| BMI <25 | **1.93 (1.74-2.14)** | **1.92 (1.67-2.20)** | **1.93 (1.38-2.69)** | **2.38 (1.61-3.53)** | **1.77 (1.33-2.35)** | **2.13 (1.80-2.52)** | **1.86 (1.30-2.64)** | **1.76 (1.22-2.53)** |
| BMI 25-<30 | **1.45 (1.28-1.64)** | **1.46 (1.23-1.72)** | **1.57 (1.03-2.39)** | **2.23 (1.47-3.39)** | 1.09 (0.77-1.52) | **1.79 (1.48-2.17)** | 1.60 (0.98-2.61) | 1.36 (0.85-2.17) |
| BMI ≥30.0 | **1.49 (1.28-1.74)** | **1.51 (1.24-1.85)** | 0.96 (0.52-1.75) | **2.78 (1.53-5.05)** | 1.17 (0.76-1.82) | **1.50 (1.18-1.90)** | 1.29 (0.65-2.55) | 1.57 (0.88-2.81) |
| **Duration of use, years** | |  |  |  |  |  |  |  |
| **<5** |  |  |  |  |  |  |  |  |
| BMI <25 | **1.41 (1.24-1.61)** | **1.41 (1.18-1.67)** | **1.63 (1.08-2.46)** | **1.78 (1.10-2.90)** | 1.08 (0.73-1.60) | **1.46 (1.17-1.80)** | 1.32 (0.83-2.09) | 1.48 (0.94-2.32) |
| BMI 25-<30 | 1.10 (0.94-1.30) | 1.02 (0.82-1.28) | 1.17 (0.67-2.04) | 1.65 (0.98-2.79) | 0.90 (0.59-1.39) | 1.14 (0.88-1.48) | 1.14 (0.61-2.15) | 1.65 (0.98-2.78) |
| BMI ≥30.0 | 1.09 (0.89-1.32) | 1.06 (0.82-1.37) | 0.91 (0.45-1.83) | 1.61 (0.74-3.53) | 0.91 (0.53-1.58) | 0.98 (0.72-1.34) | 1.07 (0.47-2.44) | 1.02 (0.48-2.14) |
| **5-9** |  |  |  |  |  |  |  |  |
| BMI <25 | **1.74 (1.52-1.98)** | **1.70 (1.43-2.03)** | **1.78 (1.17-2.73)** | **1.89 (1.14-3.13)** | 1.38 (0.94-2.03) | **1.71 (1.38-2.13)** | **1.82 (1.17-2.85)** | **1.83 (1.16-2.87)** |
| BMI 25-<30 | **1.59 (1.35-1.86)** | **1.57 (1.26-1.95)** | **1.89 (1.11-3.22)** | **1.99 (1.15-3.45)** | 1.30 (0.84-2.02) | **1.9 (1.48-2.43)** | 1.50 (0.78-2.88) | 1.59 (0.87-2.90) |
| BMI ≥30.0 | **1.64 (1.32-2.02)** | **1.77 (1.35-2.31)** | 0.69 (0.24-1.93) | **2.66 (1.20-5.88)** | 1.18 (0.63-2.22) | **1.84 (1.35-2.51)** | 1.16 (0.40-3.35) | **2.26 (1.10-4.66)** |
| **≥10** |  |  |  |  |  |  |  |  |
| BMI <25 | **2.27 (2.00-2.58)** | **2.36 (2.00-2.79)** | **1.71 (1.09-2.68)** | **2.96 (1.86-4.71)** | **2.44 (1.74-3.42)** | **2.89 (2.37-3.52)** | **2.18 (1.40-3.40)** | **1.70 (1.04-2.77)** |
| BMI 25-<30 | **1.58 (1.33-1.88)** | **1.67 (1.33-2.10)** | 1.71 (0.96-3.02) | **2.94 (1.73-4.99)** | 0.98 (0.57-1.68) | **2.18 (1.70-2.79)** | 1.64 (0.82-3.27) | 1.22 (0.59-2.53) |
| BMI ≥30.0 | **1.62 (1.28-2.06)** | **1.59 (1.16-2.19)** | 0.91 (0.32-2.56) | **3.72 (1.68-8.21)** | 1.57 (0.85-2.89) | **1.77 (1.23-2.54)** | 1.36 (0.47-3.91) | 1.95 (0.82-4.68) |

MHT, menopausal hormone therapy; IDC, invasive ductal carcinoma; ILC, invasive lobular carcinoma; ER, estrogen receptor; PR, progesterone receptor

Bold indicates p value< 0.05.

**Table S7**

| **Hormone therapy** | **Breast cancer subtype by hormone receptor status  (HR, 95%CIs)** | | | | |  |
| --- | --- | --- | --- | --- | --- | --- |
|  | **ER+PR+** | **ER+PR-** | | **ER-PR-** | |  |
| **No. of cases** |  | |  | |  | |
| Total breast cancer | 2022 | | 374 | | 398 | |
| Invasive ductal cancer | 1440 | | 241 | | 320 | |
| Invasive lobular cancer | 247 | | 61 | | 15 | |
| **Never MHT** | 1.00 referent | | 1.00 referent | | 1.00 referent | |
| **Ever EPT use** |  | |  | |  | |
| Total breast cancer | **1.67 (1.51-1.85)** | | **1.54 (1.21-1.97)** | | **1.57 (1.24-1.99)** | |
| Invasive ductal cancer | **1.63 (1.44-1.84)** | | **1.58 (1.17-2.14)** | | **1.63 (1.25-2.13)** | |
| Invasive lobular cancer | **1.99 (1.48-2.68)** | | 0.61 (0.31-1.23) | | 0.58 (0.14-2.38) | |
| **Former user** |  | |  | |  | |
| Total breast cancer | 1.15 (0.94-1.40) | | 0.90 (0.54-1.49) | | 1.46 (0.98-2.18) | |
| Invasive ductal cancer | 1.12 (0.89-1.42) | | 0.96 (0.53-1.76) | | **1.64 (1.07-2.52)** | |
| Invasive lobular cancer | 1.41 (0.82-2.41) | | - | | - | |
| **Current user** |  | |  | |  | |
| Total breast cancer | **1.83 (1.64-2.04)** | | **1.71 (1.33-2.21)** | | **1.58 (1.23-2.04)** | |
| Invasive ductal cancer | **1.79 (1.57-2.03)** | | **1.73 (1.26-2.37)** | | **1.62 (1.22-2.14)** | |
| Invasive lobular cancer | **2.18 (1.60-2.97)** | | 0.79 (0.39-1.60) | | 0.48 (0.10-2.45) | |

| **Duration of use, years** |  |  |  | | |
| --- | --- | --- | --- | --- | --- |
| **<5** |  |  |  | | |
| Total breast cancer | **1.21 (1.05-1.40)** | 1.24 (0.90-1.73) | **1.45 (1.07-1.96)** | | |
| Invasive ductal cancer | 1.17 (0.99-1.39) | 1.04 (0.68-1.61) | | **1.55 (1.11-2.17)** |  |
| Invasive lobular cancer | **1.56 (1.05-2.31)** | 0.74 (0.30-1.80) | | 0.44 (0.05-3.73) |  |
| **5-9** |  |  | |  |  |
| Total breast cancer | **1.73 (1.50-1.99)** | **1.64 (1.17-2.30)** | | **1.79 (1.30-2.46)** |  |
| Invasive ductal cancer | **1.73 (1.46-2.05)** | **1.80 (1.20-2.71)** | | **1.84 (1.29-2.62)** |  |
| Invasive lobular cancer | **1.93 (1.29-2.91)** | 0.67 (0.23-1.95) | | 0.61 (0.07-5.20) |  |
| **≥10** |  |  | |  |  |
| Total breast cancer | **2.38 (2.07-2.73)** | **1.94 (1.38-2.72)** | | **1.55 (1.08-2.23)** |  |
| Invasive ductal cancer | **2.31 (1.96-2.72)** | **2.21 (1.48-3.30)** | | **1.60 (1.07-2.39)** |  |
| Invasive lobular cancer | **2.59 (1.76-3.82)** | 0.38 (0.09-1.61) | | **-** |  |

ER, estrogen receptor; PR, progesterone receptor

Bold indicates p value< 0.05.
